# Priorities for public health preparedness for psychedelics: education, workforce readiness, and strategic information needs in a rapidly changing landscape

**DOI:** 10.3389/fpubh.2026.1900601

**Published:** 2026-08-06

**Authors:** R. Cameron Wolf, Rick Lambert, Sara Shoar, Joshua S. Hunt, Ingrid Donato, Neeraj Gandotra

**Affiliations:** 1 Substance Abuse and Mental Health Services Administration (SAMHSA), Rockville, MD, United States; 2 Keck School of Medicine, University of Southern California, Los Angeles, CA, United States

**Keywords:** emerging therapeutics, health education, psychedelics, public health, strategic information, substance use and misuse, workforce readiness

## Abstract

Use of psychedelic substances is increasing across unregulated, state-regulated, research, ceremonial/religious, and medical settings in the United States. Concurrently, a growing body of clinical research signals the potential for therapeutic applications for specific psychedelics in the treatment of depression, post-traumatic stress disorder, substance use disorders, and other health conditions. This evolving landscape presents both promise and complexity. From a public health perspective, a structured public health preparedness approach is needed to prioritize safety, workforce readiness, and data infrastructure in a manner that promotes individual and collective well-being while supporting responsible evidence development. This commentary proposes priorities for public health preparedness for psychedelics: (1) education and prevention of adverse events; (2) behavioral health workforce development; and (3) strategic information and data analysis. Educational efforts should emphasize clear risk communication, substance-specific messaging, prevention of adverse events, and practical guidance to support policy decision-making. Workforce considerations include defining core competencies, clarifying ethical boundaries and informed consent, establishing training and technical assistance infrastructure, strengthening capacity across the behavioral health workforce, and preparing providers to respond to patients using psychedelics outside regulated systems. Strategic information priorities include strengthening data systems and improving coordination and harmonization of epidemiologic monitoring related to patterns of use, adverse events, treatment-seeking behaviors, and the implications of evolving policy environments. As psychedelics enter mainstream discourse, public health institutions must adopt a balanced approach that considers both benefits and risks. A proactive, health-promoting, and data-driven framework can help ensure that policies and practices are grounded in safety and core public health principles.

## Introduction

Use of psychedelic substances, which are often grouped within broader hallucinogen categories in surveillance systems, is increasing to historically high levels across unregulated, state-authorized, research, ceremonial/religious, and medical settings in the United States (1–6). According to the National Survey on Drug Use and Health, between 2021 and 2024, past-year hallucinogen use among individuals aged 12 years and older increased 38%, from 7.5 million to 10.4 million in the United States; in 2024 alone, approximately 1.6 million individuals aged 12 years and older newly initiated use of a hallucinogenic substance (5). This was largely driven by increasing use of psilocybin mushrooms (3–7), while the use of lysergic acid diethylamide (LSD) and 3,4-methylenedioxymethamphetamine (MDMA) decreased or remained stable (5, 6). This expansion is occurring within a rapidly evolving and fragmented policy environment, as multiple states pursue legislative, regulatory, and research initiatives while federal scientific investment accelerates (8–11). At the same time, a growing body of clinical research suggests potential therapeutic applications for specific psychedelics in the treatment of depression, post-traumatic stress disorder (PTSD), substance use disorders (SUD), and other health conditions that contribute to suffering and disability (12–15). Together, these developments demonstrate that psychedelics are becoming increasingly integrated into emerging health and public discourse. These trends suggest that public health and healthcare systems may increasingly encounter psychedelic-related care needs, including patient education, adverse event management, clinical consultation, care coordination, and workforce training requirements.

At the federal level, recent policy actions aimed at accelerating treatments for serious mental illness—including directives to prioritize regulatory review, expand access pathways for investigational therapies, and increase coordination across agencies—reflect growing momentum in the development of psychedelic and related compounds (16). On April 18th, 2026, President Trump issued an Executive Order titled *Accelerating Medical Treatments for Serious Mental Illness*, further underscoring federal interest in expediting drug development pathways for emerging psychiatric interventions. However, the Executive Order does not currently address parallel public health challenges related to workforce readiness, patient access, healthcare delivery and reimbursement infrastructure, or long-term monitoring systems. These efforts have been rapidly operationalized through the use of expedited regulatory mechanisms. For example, the United States’ Food and Drug Administration (FDA) has recently issued priority review vouchers to multiple psychedelic drug development programs, with the potential to substantially shorten review timelines for qualifying applications (17). These developments suggest that the timeline between research, policy development, and potential clinical availability is sharply narrowing.

The evolving landscape presents both promise and complexity. These dynamics are unfolding more quickly than the development of corresponding public health infrastructure. Existing public health and behavioral health systems, workforce capacity, and epidemiologic data infrastructure were not designed with these substances in mind.

From a public health perspective, this moment represents a critical need for a structured, forward-looking vision that emphasizes safety, preparedness, and evidence-informed decision-making. Public health institutions are uniquely positioned to support coordination across clinical, community, and policy domains, while maintaining a focus on individual and population-level well-being. Comprehensive public health frameworks encompass a broad range of essential public health functions. This Perspective focuses specifically on three preparedness domains that we believe warrant particular attention as psychedelic-related policies, services, and patterns of use continue to evolve: (1) education and prevention of adverse events; (2) behavioral health workforce development; and (3) strategic information and data analysis. These three preparedness domains complement broader public health frameworks, including the CDC’s 10 Essential Public Health Services, by highlighting preparedness priorities particularly relevant to the evolving psychedelic landscape (18, 19). The domains are not intended to endorse specific policy models or clinical applications, but rather to outline core public health capacities needed to prepare for a rapidly evolving landscape.

This framework was informed by the authors’ experience in public health, behavioral health policy, epidemiology, workforce development, and emerging discussions regarding psychedelic-related services. It also draws upon current scientific literature, evolving policy environments, and public health preparedness principles.

### Psychedelics as a distinct public health challenge

Psychedelics present characteristics that distinguish them from many other substances encountered within health systems, introducing unique considerations for public health planning.

First, psychedelic substances are associated with intense and time-limited alterations in perception, cognition, and emotion, often lasting several hours (20). Outcomes can vary widely depending on dose, individual characteristics, and context. While adverse events are relatively uncommon in controlled settings, they can be acute and require immediate response (21) and may occur more frequently in unregulated use environments (22–24). Given the extended duration of psychoactive effects for many psychedelics, individuals may also require supportive services, including transportation and monitoring, that may not typically be necessary for other therapeutic modalities.

Second, outcomes are often context-dependent, influenced by expectations, environment, and interpersonal dynamics (25). This increases the importance of preparation, supervision, and follow-up care, even outside formal clinical settings.

Third, the current landscape reflects a blurring of boundaries across settings. Psychedelics are used in clinical research studies, state-regulated programs, informal or unregulated environments, and ceremonial or religious contexts, including some operating under legal protections associated with religious freedom frameworks (3). These pathways are developing simultaneously without consistent standards or coordination, and individuals may move across settings.

Fourth, there is a rapid emergence of new service models and workforces, including facilitators and other non-traditional providers. Roles, competencies, and oversight mechanisms vary widely, raising questions about safety, ethics, and integration with healthcare systems (26).

Finally, scientific development is expanding beyond classic psychedelic compounds to include other novel rapid-acting neuroplasticity-promoting agents, potentially with different experiential and delivery profiles (27, 28). As federal initiatives accelerate development and regulatory review, public health systems may need to adapt to multiple interventions.

These features suggest that existing public health tools—developed for more conventional substances and care models—will require adaptation.

### Priorities for public health preparedness

#### Pillar 1: education and prevention of adverse events

Education is a foundational public health strategy and is particularly important in the context of psychedelics, where public understanding is shaped by a mix of scientific evidence, media narratives, and informal sources.

A central objective is to support the prevention of adverse events through clear communication about risks, contraindications, and factors associated with negative outcomes. This includes conveying variability in experiences, identifying populations at increased risk (e.g., individuals with certain psychiatric or medical conditions), and highlighting potential interactions with medications (29, 30). Potential adverse events may include acute psychological distress, prolonged perceptual or emotional disturbances, unsafe behaviors occurring during intoxication, and challenges associated with delayed recognition of adverse reactions.

Educational strategies should be substance-specific given differences in pharmacology, duration, and risk. Generalized messaging may obscure important distinctions. Potency and purity (e.g., adulteration) are key factors determining potential risks.

Educational efforts must also reflect the reality that some individuals use psychedelics outside regulated settings. Providing accurate, evidence-informed risk and prevention information can help reduce negative outcomes without promoting use. Messaging should emphasize informed decision-making, awareness of high-risk situations, and when to seek care.

Audience segmentation is critical. Public messaging, education, and policy guidance should be tailored to distinct community needs (e.g., veterans, adolescents and young adults, individuals with serious mental illness, healthcare providers, and policymakers) (31). Education of stakeholders also plays a role in supporting policy deliberation, ensuring decisions are informed by balanced evidence.

#### Pillar 2: behavioral health workforce development

The evolving landscape has significant implications for the behavioral health workforce and other professionals involved in healthcare and public health systems. Providers across settings are increasingly likely to encounter individuals who have used or are considering using psychedelics.

Core competencies should include:

- Screening for risk factors (e.g., psychiatric history, medications).
- Recognizing and responding to acute and longer-term adverse effects.
- Providing follow-up care and referrals.

Providers should be equipped for person-centered care to establish a therapeutic alliance, a collaborative and trust-based relationship between patient and provider, in order to facilitate intention setting, personal disclosure, and informed conversations about potential risks and benefits. Ethical considerations are particularly important. Psychedelic experiences may involve heightened vulnerability, raising important ethical considerations related to professional boundaries, including around the use of physical touch, informed consent, and scope of practice (32, 33). Clear guidance is needed to support safe and ethical care and patient protection.

Emerging service models introduce complexity. Non-traditional providers (e.g., trained veteran peers) may play roles in some settings, raising questions related to credentialing, supervision, integration with healthcare systems, and quality assurance.

Workforce readiness extends beyond behavioral health specialists. Emergency departments, crisis systems, first responders, primary care providers, and public health professionals may encounter individuals experiencing adverse effects or seeking information regarding psychedelic use. Baseline awareness and response capacity across systems are essential.

Ongoing training and technical assistance will be necessary, particularly as accelerated research and regulatory pathways shorten the timeline between clinical development and broader demand for access to services.

#### Pillar 3: strategic information and data analysis

Robust data systems are essential for understanding patterns of use, identifying emerging risks, and informing policy decisions. Current data efforts remain fragmented, with gaps in real-time monitoring and cross-state comparability (4).

Key domains for epidemiologic monitoring include:

- Patterns of use.
- Adverse events.
- Treatment-seeking behaviors.
- Outcomes across settings.

Understanding healthcare interactions following psychedelic use can inform system planning and service needs. There is also a need to assess policy impacts, as states adopt varied approaches (10). Comparative analysis can support continuous learning and inform refinement of emerging models of care. Strengthening data infrastructure may involve:

- Enhancing epidemiologic monitoring through population-level data.
- Improving coordination of reporting across agencies and systems.
- Developing standardized definitions and harmonizing reporting tools.
- Improving specificity in surveillance and diagnostic coding to distinguish psychedelic substances from other hallucinogens with different pharmacologic and clinical profiles.
- Triangulating data through targeted analyses.

Integration with existing systems (e.g., national surveys, emergency department data, and poison control systems) can support routine epidemiologic monitoring, while targeted studies may help address critical data gaps related to emerging behavioral patterns. Examples may include integration of data from national population surveys, poison center surveillance, emergency department monitoring systems, and state-regulated psychedelic service programs. As developments accelerate, timely data use becomes increasingly important to support evidence-informed responses.

**Figure 1:**
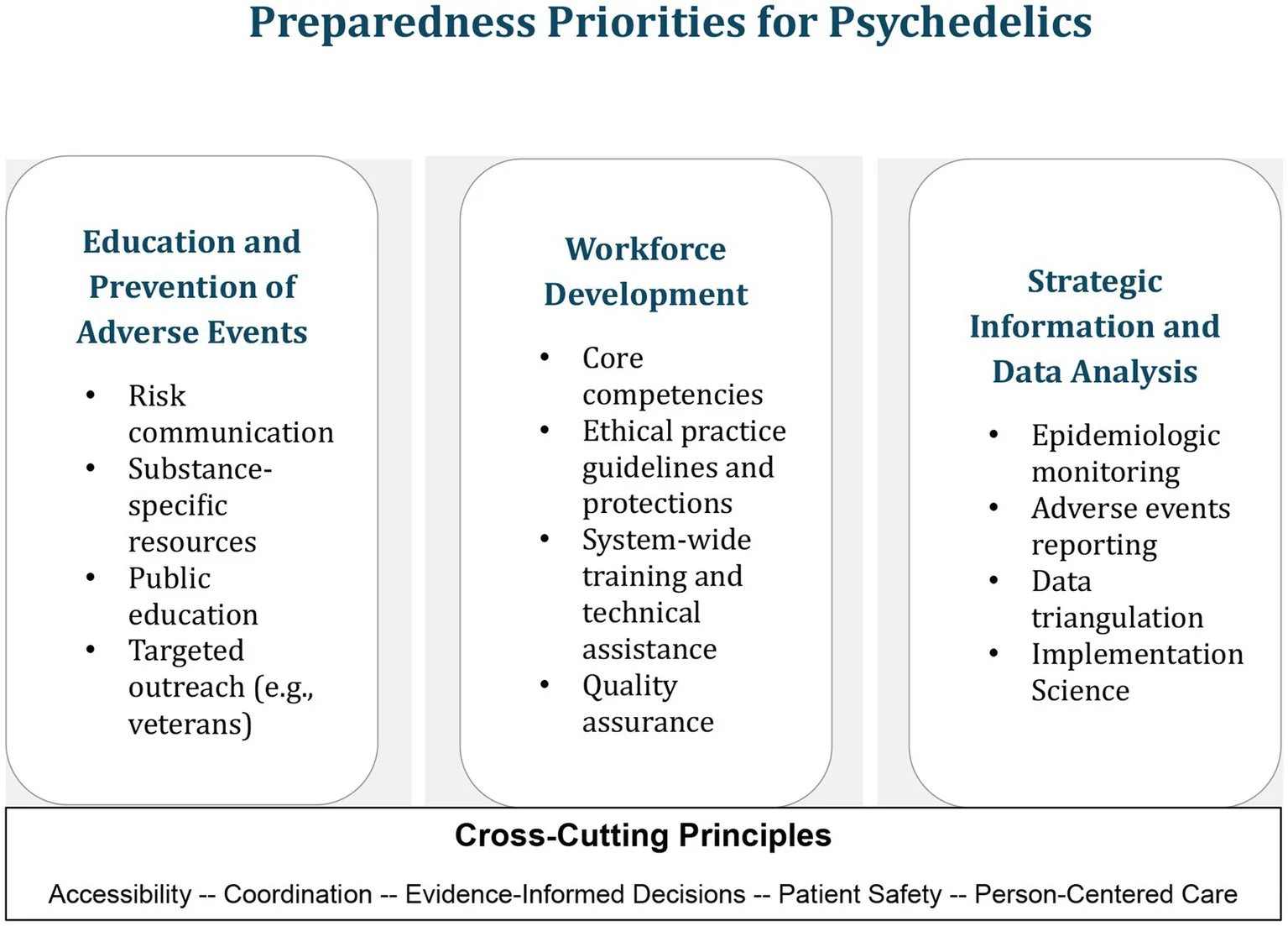
Preparedness priorities for psychedelics. The figure illustrates three priority domains for public health preparedness for psychedelics: education and prevention of adverse events, workforce development, and strategic information and data analysis. Cross-cutting principles include accessibility, coordination, evidence-informed decision-making, person-centered care, and patient safety.

Figure 1 illustrates three preparedness priorities for psychedelics, organized around the three core pillars discussed above: education, workforce development, and strategic information. Preparedness is not about a single intervention, but a coordinated system approach that includes risk communication, workforce capacity, and data-informed decision-making. The inclusion of cross-cutting principles—accessibility, coordination, evidence-informed decision-making, person-centered care, and patient safety—reinforces the need for a balanced, public health stance.

## Discussion

### Implications for public health systems

The new landscape on psychedelics underscores the need for proactive public health engagement. Ideally, systems should anticipate needs when possible rather than respond reactively. Priorities include supporting states with guidance and technical assistance, strengthening workforce capacity, and enhancing data systems. These priority domains are intended to complement broader public health frameworks and should be viewed as foundational preparedness considerations rather than a comprehensive description of all public health functions.

Public health systems have previously adapted to emerging behavioral health interventions through the development of new workforce models, clinical infrastructure, monitoring approaches, and implementation frameworks, including the expansion of new treatment, delivery and workforce models for opioid use disorder. Similar adaptive capacity may be necessary as psychedelic-related services and interventions continue to evolve. Existing public health infrastructure platforms, including integrated community behavioral health delivery systems, may offer opportunities for future implementation, evaluation, coordination, and monitoring of emerging treatment models.

The evolving psychedelics landscape also represents a broader test of how public health systems respond to rapidly emerging interventions spanning clinical and non-clinical settings. Accelerated research investment and regulatory pathways may shorten the timeline between clinical development and clinical practice, underscoring the need for preparedness. Public health programs must also consider access, affordability, and risk exposure across populations and communities. As regulated and unregulated psychedelic markets evolve, geographic differences may emerge in access to evidence-informed care, workforce availability, and protection from unsafe practices.

## Conclusion

As psychedelics gain prominence in mainstream discourse, public health institutions face a complex and evolving landscape. A balanced and transparent approach that considers both potential benefits and risks while remaining grounded in emerging evidence is essential. By focusing on education and prevention of adverse events, workforce readiness, and strategic data capacity, public health systems can support informed decision-making and promote safety. The evolving psychedelics landscape may also serve as an early test case for how public health systems respond to rapidly emerging behavioral health interventions that span medical, community, and nontraditional settings. A proactive, coordinated, and data-driven public health framework can help ensure that policies and practices are grounded in public health principles while supporting responsible innovation.

## Data Availability

The original contributions presented in the study are included in the article/supplementary material, further inquiries can be directed to the corresponding author.
